# First meeting “Cystic echinococcosis in Chile, update in alternatives for control and diagnostics in animals and humans”

**DOI:** 10.1186/s13071-016-1792-y

**Published:** 2016-09-13

**Authors:** Cristian A. Alvarez Rojas, Fernando Fredes, Marisa Torres, Gerardo Acosta-Jamett, Juan Francisco Alvarez, Carlos Pavletic, Rodolfo Paredes, Sandra Cortés, Ministry of Health, SAG and other organizations attending the meeting, Natalia Castro, Jorge Gajardo, Isabel Bertin, Leopoldo del Barrio, Felipe Troncoso, Fabiola Faundez, Nelson Flores, Loreto Uribe Boisier, María Isabel Manriquez, Sebastian Lillo, Héctor Escobar, Alejandra Poblete, Lilia Fuentes, Iván Wainnright, Samuel Serrano, Mitzi Villalón, Marjorie Arriaza, Loreto Caldera, Annelisse Fritz, Marcela Saavedra, José Antonio Segura, Carla Barrientos, Alonso Parra, Ricardo Porcel, Verónica Segovia, Cristián Sabelle, Guido Merino, Julio Gómez, María Isabel Sánchez, Susan Miranda Vicencio, Gladys Rios Aguilera

**Affiliations:** 1 Centre for Animal Biotechnology, The University of Melbourne, Parkville, 3052 Victoria Australia; 2 Departamento de Medicina Preventiva Animal Facultad de Ciencias Veterinarias y Pecuarias, Universidad de Chile, Santiago, Chile; 3 Facultad de Medicina, Pontificia Universidad Católica de Chile, Santiago, Chile; 4 Instituto de Medicina Preventiva Veterinaria y Programa de Investigación Aplicada en Fauna Silvestre, Facultad de Ciencias Veterinarias, Universidad Austral de Chile, Casilla 567, Valdivia, Chile; 5 Servicio Agrícola Ganadero, Santiago, Chile; 6 Ministerio de Salud, Gobierno de Chile, Santiago, Chile; 7 Escuela de Medicina Veterinaria, Facultad de Ecología y Recursos Naturales, Universidad Andres Bello, Santiago, Chile; 8 Advanced Center for Chronic Diseases (ACCDiS), Facultad Medicina, Pontificia Universidad Católica de Chile, Santiago, Chile

**Keywords:** Cystic echinococcosis, *Echinococcus granulosus*, Chile, Zoonosis, Neglected disease

## Abstract

This report summarizes the outcomes of a meeting on cystic echinococcosis (CE) in animals and humans in Chile held in Santiago, Chile, between the 21st and 22nd of January 2016. The meeting participants included representatives of the Departamento de Zoonosis, Ministerio de Salud (Zoonotic Diseases Department, Ministry of Health), representatives of the Secretarias Regionales del Ministerio de Salud (Regional Department of Health, Ministry of Health), Instituto Nacional de Desarrollo Agropecuario (National Institute for the Development of Agriculture and Livestock, INDAP), Instituto de Salud Pública (National Institute for Public Health, ISP) and the Servicio Agrícola y Ganadero (Animal Health Department, SAG), academics from various universities, veterinarians and physicians. Current and future CE control activities were discussed. It was noted that the EG95 vaccine was being implemented for the first time in pilot control programmes, with the vaccine scheduled during 2016 in two different regions in the South of Chile. In relation to use of the vaccine, the need was highlighted for acquiring good quality data, based on CE findings at slaughterhouse, previous to initiation of vaccination so as to enable correct assessment of the efficacy of the vaccine in the following years. The current world’s-best-practice concerning the use of ultrasound as a diagnostic tool for the screening population in highly endemic remote and poor areas was also discussed.

## Background

**Fig. 1:**
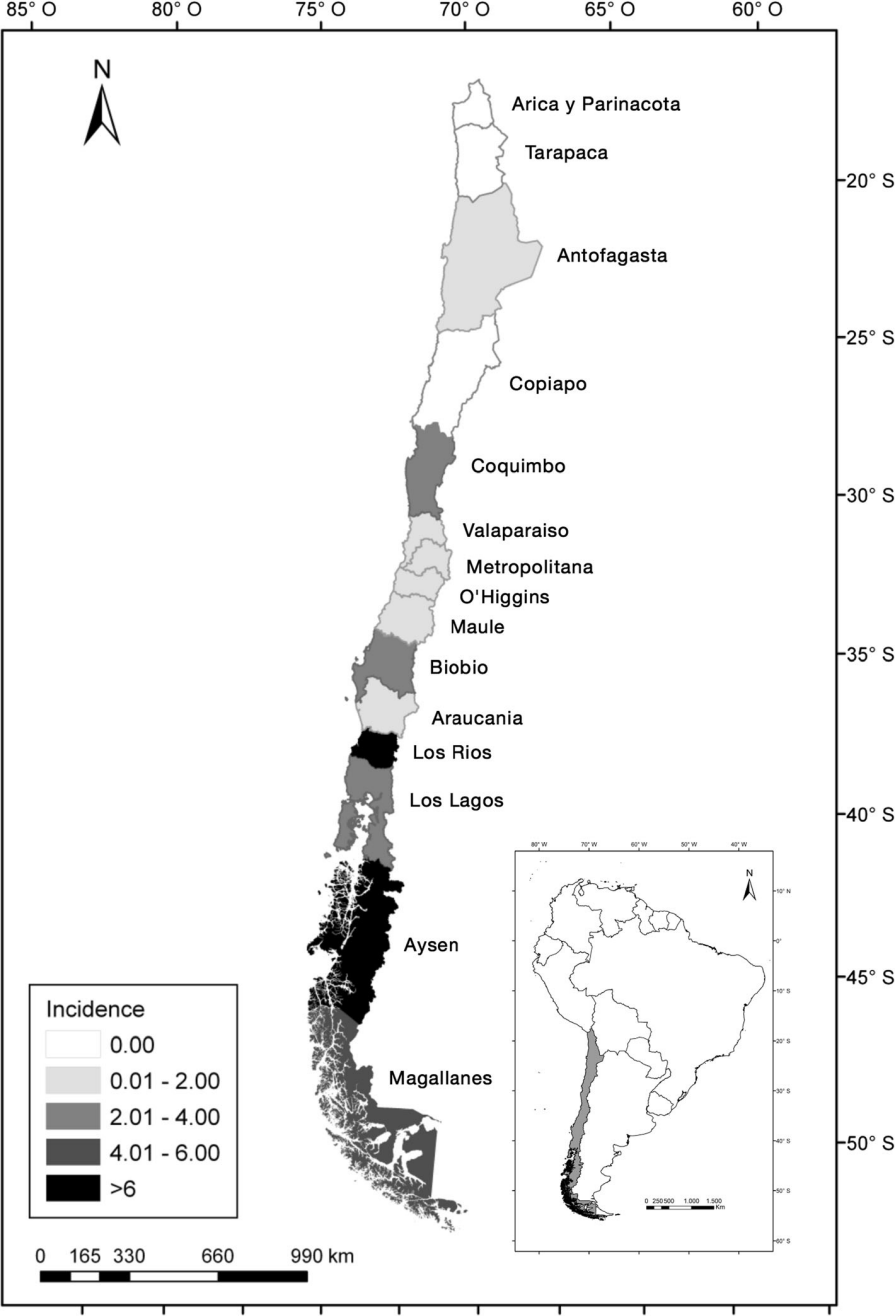
Map of Chile showing its administrative division in fifteen regions with their respective names. Grayscale colours indicate the incidence of human cystic echinococcosis per 100,000 based on data from 2015

Cystic echinococcosis (CE) remains as an important health problem affecting animals and humans in Chile [1, 2]. A variety of biogeographic areas are present in Chile in a long and narrow strip of land (4,300 km long). The country is divided into fifteen administrative regions, including the capital, Santiago, which is usually referred as “Region Metropolitana” (RM). Currently, the estimated incidence for CE in humans in Chile is 1.4–1.8/100,000 [2] (see Fig. 1 for a detailed explanation of prevalence by region). However, based on CE cases discharged from hospitals the incidence raises to numbers between 4.7–5.0/100,000 indicating an important level of underreporting of the disease in Chile [2]. In the case of livestock animals, *Echinococcus granulosus* is the second most important cause of condemnation of viscera in abattoirs in Chile, after *Fasciola hepatica*. Data from 2014 acquired at official abattoirs show a national CE incidence of 305/1,000 animals for goats; 200 cases/1,000 animals for cattle; 31/1,000 for sheep; 12/1,000 animals for horses; for porcine hosts the rate is lower than 1 case/1,000 animals [3]. The most affected regions are Los Lagos, Araucania, Los Rios, Aysen and Magallanes [2]. Regarding dog infections with *E. granulosus*, the information is scarce with no data available at a national level. An arecoline-purge survey of 1,358 dogs showed an infection of 11 % in 2000 in El Maule region (central area of Chile) [4], while another arecoline survey in 228 dogs from Magallanes region (southern area of Chile, under control) found a prevalence of canine echinococcosis of 1.8 % in 2001 [5]. More recently, 13.6 % of dog faeces from 596 farms were positive using copro-PCR in Lonquimay (Araucania region) [6]. Another study performed in Coquimbo region (northern area of Chile) in 2014, showed a high prevalence in dogs from rural and urban areas (between 3.5–11.7 %) using coproantigen test [7]. A second study, using similar methodology, showed a higher rate of infection, 28 % in rural areas of five municipalities from the Coquimbo region [8]. In terms of molecular studies on *E. granulosus* isolates in Chile, a single case of human infected with the genotype G6 has been reported in a study in which 19 other human samples were found to be *E. granulosus* (*sensu stricto*) (*s.s*.) [9]. A second study confirmed the presence of *E. granulosus* (*s.s*.) in cattle from the South of Chile [10].

At the moment there is no official national programme for the control of *E. granulosus*. However, local control plans (based on specific regions) have been undertaken intermittently during the last 30 years, financially supported with national and regional funds. One of the most relevant and successful local control programmes was developed in the Magallanes region between 1979–2004. The area is characterized by large population of sheep, numbering 2,205,477 in 2007. During the time the control plan was in action it was possible to achieve a significant reduction of the sheep infection from 60 % in 1978 to 0.7 % in 2004 and in dogs from 71.4 to 0.5 %, respectively [5, 11]. Although the programme was highly successful, it was interrupted in 2004 and there was no plan implemented to maintain the low prevalence achieved. Since 2015 four different control programmes have been initiated in different regions of the country: Coquimbo, Bio Bio, Araucania and Aysen. Some of these initiatives include pilot sheep vaccination trials with the commercial version of the EG95 vaccine planned to be implemented in 2016.

## Objectives of the meeting

**Fig. 2:**
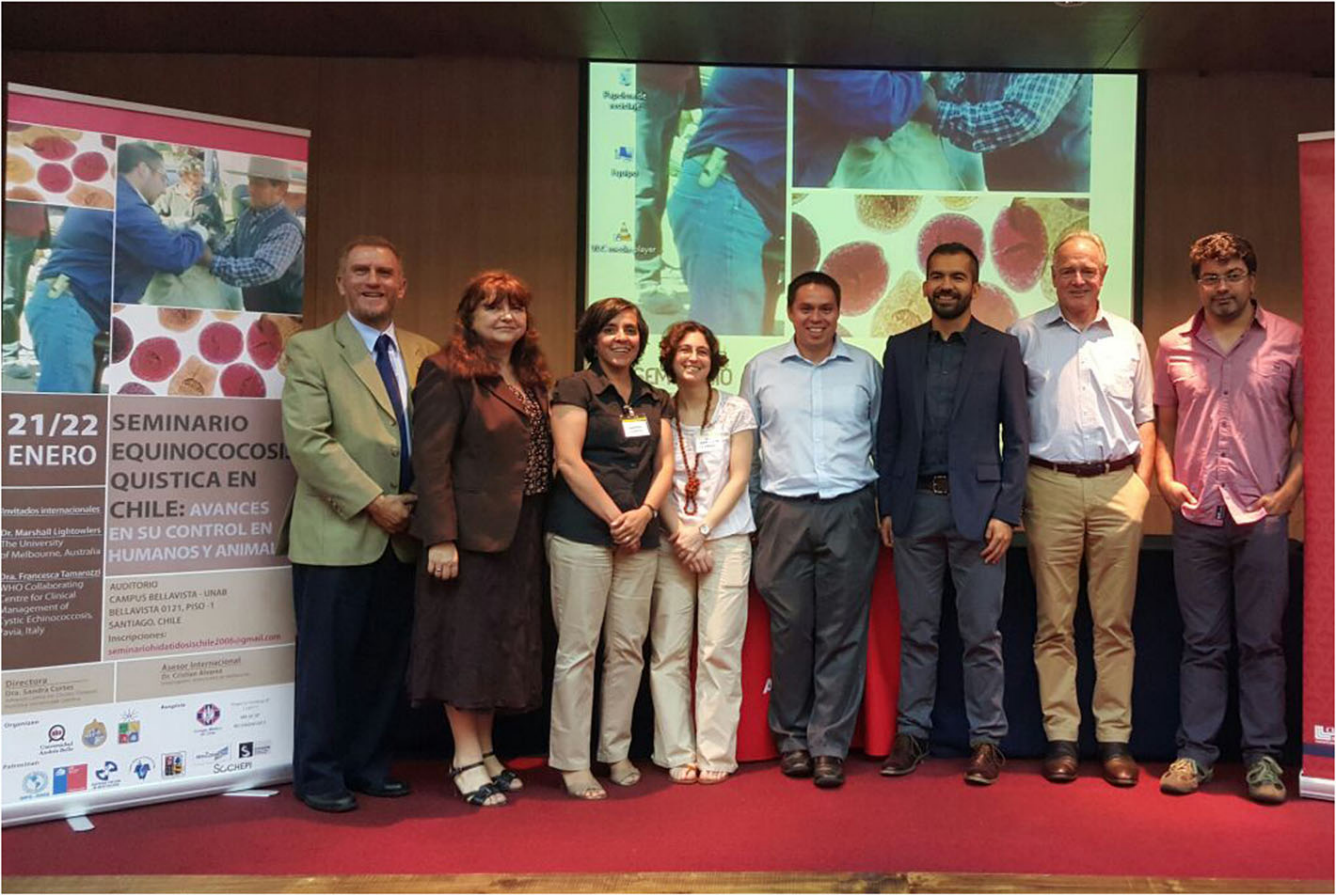
Members of the organizing committee of the first meeting “Cystic echinococcosis in Chile, update in alternatives for control and diagnostics in animals and humans”. From left to right: Dr. Fernando Fredes, Dr. Marisa Torres, Dr. Sandra Cortes, Dr. Francesca Tamarozzi (International speaker), Dr. Rodolfo Paredes, Dr. Cristian A. Alvarez Rojas, Professor Marshall Lightowlers (International speaker) and Dr. Gerardo Acosta-Jamett

The new initiatives for the control of CE that started in 2015 motivated a group of scientists in Chile having a special interest in CE to organize a meeting related to the control of this disease. The principal objective of the meeting was to bring together the different groups working on CE in Chile, especially those working on control of the transmission of the disease, to share information, experience and to enhance collaboration. The timely visit of one of the international speakers, Melbourne Laureate Professor Marshall Lightowlers, in the year in which the vaccine EG95 was thought to be in use in pilot activities in some regions in Chile, was a perfect opportunity to reinforce the achievements of the objectives proposed by us, including: (i) Provide a forum in which local authorities in charge of control programmes could exchange their knowledge of the epidemiology of the disease with the scientific community, other governmental institutions and with professionals and students of health related professions; (ii) Academics could update current knowledge and world’s-best-practice concerning *E. granulosus* control tools (e.g. aspects concerning the application of anthelmintics in dogs and use of the EG95 vaccine in livestock) and the use of ultrasound as a diagnostic tool for the screening population in highly endemic remote and poor areas; (iii) Establish a study group for cystic echinococcosis in Chile. Figure 2 shows the members of the organizing committee plus the international speakers Professor Marshall Lightowlers and Dr. Francesca Tamarozzi. The meeting was organized in four main sections which are described below together with highlights of each session.

### Section 1: General aspects of the epidemiology of *E. granulosus* in Chile

Dr. Carla Barrientos (Ministry of Health of Chile) presented epidemiological data concerning the current situation of human CE in Chile, including incidence and mortality rates and also highlighted the issue of under-reporting of cases. Dr. Barrientos also commented in the large dog population in the country estimated between 3 to 3.5 million dogs. The current control CE programmes that are initiatives of the Ministry of Health of Chile were described, including control programmes in the regions Coquimbo, Bio Bio, Araucania, Aysen and Magallanes, plus a control campaign in San Pedro de Melipilla (RM) (Fig. 1). The highest human incidence is reported in the Aysén region (36.7/100,000 inhabitants), followed by region XII, Magallanes (8.7/100,000 inhabitants), Araucania (8.4/100 inhabitants), BioBio (3.6/100,000 inhabitants), Los Lagos (3.2/100,000 inhabitants) and Coquimbo (2.6/100,000 inhabitants). Dr. Barrientos also described the legal framework in which the notification of human and animal disease is compulsory and discussed the challenges for the future, including the maintenance of the control initiatives, the extent of this initiatives to other parts of the country, the need for reinforcing the intersectoral collaboration between the Ministry of Health, Agriculture, councils and local communities and finally, to facilitate the active involvement of the community.

Dr. Juan Francisco Álvarez (Servicio Agrícola Ganadero) presented a detailed historical perspective of CE control programmes carried out in the Magallanes region of Chile between 1979–2004 aimed to decrease the infection levels from 60 % in sheep to under 10 % in the first 10 years and under 3 % in the subsequent years. The main strategy of the control programme was the use of praziquantel together with register of dogs, epidemiological surveillance (presence of the parasite in dog faeces and sheep infection), sanitary control (control of entry of new dogs to the region, building of infrastructure for local sacrifice of animals, in areas where there is no official abattoir, correct disposal of viscera), public health education (avoid feeding of dogs with offal, understanding of the life-cycle of the parasite through local radio and schools), capacity building (laboratory facilities for diagnostic, training of staff in diagnostic techniques), and finally the modification and creation of laws and regulations that will sustain the programme. The last treatment of dog was performed in 2004; since then an increase in the infection in sheep from 0.73 % in 2004 to 2.64 % in 2015 has been observed.

Dr. Marisa Torres (Pontificia Universidad Católica) described the importance of CE affecting humans and how it continues to being a neglected issue in Chile. Dr. Torres also described how socioeconomic, cultural and environmental conditions facilitate the transmission of this parasite with emphasis on Chile. Special attention was devoted to the lack of knowledge of this parasite and its transmission cycle among the farmers which continue to feed dogs with offal and also the lack of anti-parasitic treatment to dogs. Dr. Torres also highlighted how the poor communities, where the disease is highly prevalent, are neglected and that usually the diagnosis of CE is late when is already compromising the wellbeing of patients. Dr. Torres emphasised the lack of standardization of diagnostic methods, especially related with protocols for CE diagnostic and categorization of the disease using ultrasound and stressed the need for creating a national control plan integrating animal and human health.

Ms. Maria Paz Aylwin (Instituto de Salud Pública (ISP), National Reference Laboratory for Parasitology) summarized the diagnostic activities carried out at the reference laboratory in Chile. The ISP counts with an in house made ELISA IgG and a Western blot used for monitoring the antibody concentration pre- and post-surgical treatment of patients. The ISP also provides copro PCR diagnostic service amplifying sections of *cox1* and 12S rRNA genes. The lab has been training people coming from endemic countries in South America in molecular diagnostics as part of the Initiative from the control of CE supported by PAHO.

#### Highlights

Although CE receives attention by the authorities in Chile, the disease persists and is an important neglected public health issue affecting many people. The true extent of the disease burden in the human population in Chile due to CE remains unknown because of an under-reporting of surgical cases. Challenges that remain for the future include: the sustainability of the initiatives of control into the future, expansion of control programmes to the whole country, improving and incentivising intersectoral work and increasing the involvement of the community in these initiatives. Regarding control programmes especially in the southern region of the country (Magallanes), after 25 years of a successful control programme implementation that decreased substantially the infection rates in sheep and dogs it has been observed an increase in the infection in sheep from 0.73 % in 2004 to 2.64 % in 2015 (J.F. Alvarez, personal communication).

### Section 2: Current local and regional experiences in CE control in Chile

Mr. Oscar Aguirre (Anthropologist representing the Department of Epidemiology of the Ministry of Health in the O’Higgins region) summarised the activities carried out in the region during the period 2008–2015. The control programme currently underway in this region is based on educational activities. The programme comprises eight municipalities with a total population of 59,394 inhabitants and includes training people in control activities; people attending the training sessions come from different small organizations including mostly education (schools), and health related organizations and neighbourhood associations. In total, 8,909 students from primary and secondary schools have received instructions in prevention of CE in periodic visits by the team of the Epidemiology Department of the O’Higgins region. Mr. Aguirre described in detail these activities and showed a number of images of the training sessions and visits to schools in the region and also posters summarizing the prevention measures for the infection with the parasite. Mr. Aguirre analysed the challenges for the implementation of the programme. Since the programme has been based on education of the population in prevention of the disease, there is still a need to identify the infected cases, treat them and maintain a surveillance of the disease. It is expected the programme will include one of the regional diagnostic laboratories for the screening of the population. Another challenge is the inclusion of more municipalities in the programme and the inclusion of a qualitative assessment of the measures implemented.

Dr. Maria Isabel Manriquez (Ministry of Health) presented the situation of CE in Araucanía region. In this region an interdisciplinary committee was established in 2010; this committee proposed a control programme involving a baseline study for *E. granulosus* prevalence in dogs between 2011–2012, followed by deworming of dogs every two months in an urban area and every four months in the rural areas. The programme has focussed on what is referred to as a cultural approach with the inclusion of representatives of the municipalities, INDAP, SAG and community leaders all directed from the regional section of the Ministry of Health. The results reported a baseline of 13.6 % of canine infection in 2012 that decreased to 4.6 % in Lonquimay area in 2014 and to 1.2 % in rural areas in 2015. The programme has included a number of activities with the Pehuenche indigenous community. Critical points identified in this presentation included: the lack of inclusion of the contents of the programme in classrooms in primary and secondary schools, lack of regulations of dog ownership, few alternatives for elimination of viscera, lack of programmes for spaying/neutering of dogs and failing in the early diagnosis of human CE cases.

Dr. Claudia Adones (Ministry of Health) presented insights of the control programme in the Coquimbo region, particularly in the councils of Punitaqui, Monte Patria and Combarbala as part of a control programme extending from 2015 to 2017. The programme involves scientists from the Universidad Católica del Norte and the Universidad Austral de Chile. The Universidad Católica del Norte is delivering an educational programme for school children and is also producing and disseminating information related to CE prevention using local radio and TV stations. The Universidad Austral de Chile is responsible for the maintenance of a register of the dog population, deworming and spaying/neutering of dogs and the assessment of *E. granulosus* infections in canids pre- and post-intervention strategies. The human population that benefit with the control programme comprises 30,914 inhabitants. The dog population being treated and registered is estimated to be between 15,425 and 28,045 dogs. The commitment of the municipalities and communities and staff involved in the programme was considered to be a particular strength of the programme. Difficulties caused by heavy rains and unpredictable natural events such as earthquakes that are commonly occurring in Chile have affected the delivery of the scheduled activities.

Dr. Sebastian Lillo (member of a governmental institution involved in the development of the agriculture in Chile with emphasis on small producers, INDAP) presented a summary of the situation in the Biobio region (Concepcion). The beneficiaries of INDAP are usually people of advanced age with a low educational level and a low financial income. Usually they have a small number of animals that are part of an inefficient production system with most of production being for self-consumption. The presentation focussed on the difficulties regarding the implementation of control measures in this environment and also in how complex it is to change habits that perpetuate the cycle.

#### Highlights

This session was extremely informative and highlighted the substantial current efforts and commitment of the professionals from the Ministry of Health and the Ministry of Agriculture working in the affected regions in attempts to control CE. However, there is an agreement of a lack of central coordination of the programmes. In terms of new data, the presentations in this section provided information on the current prevalence of dog infections in the different areas of the country; these data are yet to be published in the scientific literature.

### Section 3: Contributions of Chilean scientists to the control, biology and epidemiology of *E. granulosus*

Dr. Cristian Alvarez (University of Melbourne, Australia) presented new unpublished data on the molecular epidemiology of *E. granulosus* (*s.s*.) in Chile, describing a higher variability than previously known, based on the sequencing of 1,609 bp of the *cox1* gene from isolates from cattle, sheep and humans in a wide geographical area. The importance of characterising *E. granulosus* isolates in animals that could harbour genotypes of the parasite that could affect humans, especially goats and pigs for the G6/7 variant of *E. canadensis*, which has been found in around 10 % of the human cases of CE worldwide [12]. Previous studies by Dr. Alvarez have highlighted the need of studying the efficacy of the current EG95 vaccine against different genotypes/species of *E. granulosus*, especially those able to infect humans [13].

Dr. Rodolfo Paredes (Universidad Andres Bello) summarised his investigations on the role of the immune system in the fertility of the cysts in cattle. In the first part of his talk, Dr. Paredes showed the latest result obtained in prevalence of cystic echinococcosis in bovines slaughtered in Santiago, Chile. The unpublished data showed a large predomination of infertile cyst; 61 % of the cysts were present only in lungs, with G1 as the most important genotype present in Chile. In the second part, Dr. Paredes presented previous research, showing that germinal layer of infertile cysts presents IgG at least an order of magnitude greater than in the germinal layer of fertile cysts [14] with IgG1 being the predominant immunoglobulin subclass present in the germinal layer of infertile cysts. This suggests that bovine production of IgG1 subclass against parasite antigens present in the germinal layer could be involved in the mechanism of cyst infertility [15]. Finally, Dr. Paredes showed preliminary data on the immunological modulation in animals co-infected with *Fasciola hepatica*. At the serological level, co-infection raised the IL-4 level predominantly in infertile cyst while at adventitial layer INF-ƴ increased at least two-fold in infertile cyst.

Dr. Gerardo Acosta-Jamett (Universidad Austral de Chile) summarized his studies on the epidemiology of CE at the interphase of the urban and rural settings in the Coquimbo region and highlighted the importance of studying the dynamics of the dog population in any control intervention for this disease. The human:dog ratio in the Coquimbo region varies between 1 and 2 in rural areas and between 5 and 6 in the urban settings of the cities Coquimbo and Ovalle [16]. He also showed data referring to the presence of coproantigens in 7/28 “chilla” fox (*Lycalopex griseus*) in the Coquimbo region [17]. Human prevalence, based in serological tests, showed a rate of 2.6 % (10/403) and a coproantigen test in the same area of the Coquimbo region showed a rate of 28 % (26/93) for dogs [8].

#### Highlights

The session highlighted the efforts of three independent Chilean researchers to continue the investigations related to *E. granulosus* in three different areas: molecular epidemiology, biology and epidemiology. There was an agreement that the collaboration within Academia and between academics and the Ministry of Health, Ministry of Agriculture, Government and policy and decision makers is vital for the correct implementation of control interventions for this neglected parasite in the near future.

## Invited speakers

Melbourne Laureate Professor Marshall Lightowlers (The University of Melbourne, Australia) delivered a presentation on the “Alternatives for the control of cystic echinococcosis”, providing a historical perspective on the development of the EG95 vaccine and the contribution of numerous researchers involved in the study of CE worldwide. Together with the already known efficacy of the vaccine [18, 19], he reported the use of the vaccine in field trials in Rio Negro, Argentina, and discussed issues affecting the application of the vaccine in control programmes [20, 21], including the lack of incentive for livestock owners to vaccinate, the lack of animal handling facilities in many small farms where CE transmission is prevalent, the remote locations where control programmes are often being undertaken, as well as difficulties in the coordination of activities and the costs involved, irrespective of the cost of the vaccine itself.

Dr. Francesca Tamarozzi (University of Pavia, Italy) shared her experience in two aspects of the diagnosis of human disease: serology and ultrasonography (US). The general advantages of US are the low-cost, non-invasivity, repeatability, ad being highly accepted by the population, which is also sensitized as patients can see the cyst (if present). In diagnosis and staging of abdominal CE, US is the most accurate imaging tool compared to Magnetic Resonance Imaging and computerised tomography. Dr. Tamarozzi summarised the organization and rationale of US surveys for CE carried out in Tibet (PRC), Morocco, Perú, and most recently, within the EU “HERACLES” project in three endemic countries in eastern Europe. Important ethical issues to be considered before carrying out a population screening, as well as the importance of training of local doctors and dissemination of the results, were especially highlighted. Dr. Tamarozzi also described the WHO-IWGE (Informal Working Group on Echinococcosis) consensus classification of CE cysts and discussed the stage-specific clinical management options. In a second presentation, Dr. Tamarozzi illustrated the difficulties in the interpretation of the serology results for the diagnosis and follow-up of CE. She stressed that serology has only a complementary role to imaging, both in the context of individual patients and of prevalence studies, where current serodiagnostic tests alone are not appropriate to assess infection prevalence. Available serological tests have unsatisfactory sensitivity and specificity; heterogeneous performances and results are influenced by a number of variables, which needs to be known when interpreting results. New areas of research in this field include the use of recombinant antigens and the detection of host cytokines.

### Highlights

The presence of the invited speakers at the meeting attracted high level of interest from the audience and was a crucial factor on the success of this event. The talk by Professor Lightowlers was the first of this kind in Chile to a general audience involving not only academics but representatives of the Ministry of Health and Agriculture. Government representatives had the opportunity to hear first-hand about the development of the EG95 vaccine from a scientific and unbiased perspective. Since the usage of the vaccine will begin in some areas in Chile during 2016 in pilot trials, the timely presence of Professor Lightowlers highlighted the benefits of the inclusion of the vaccine in a control programme but also pointed out some difficulties in the practical implementation of its use, i.e. difficulty accessing animals in remote places and the lack of a commercial incentive for livestock owners to vaccinate against cystic echinococcosis, hence the critical importance of minimizing the cost of vaccination to the livestock owners. His presentation revealed that to date, after 20 years from the publication of the discovery of the EG95 vaccine, there are very limited good quality data available which assesses the real impact of the vaccine in field conditions. Therefore, the control plans developed in Chile that include vaccination are an opportunity to produce scientific evidence of the usefulness of the vaccine, so it can be implemented in other parts of the world.

The contribution of Dr. Tamarozzi was highly appreciated by the audience, she pointed out the need to establish ultrasound diagnostic in endemic poor areas. This is not the case of Chile, country in which there are no systemic data of mass ultrasound screening. Another interesting situation highlighted by Dr. Tamarozzi was the need for training of local doctors for the correct interpretation of ultrasound scans. The presence of Dr. Tamarozzi made evident a clear problem in Chile, i.e. the need for increased collaboration between physicians and veterinarians in the field of parasitology in general and especially in the area of cystic echinococcosis. Among the few physicians attending the meeting it was possible to discuss the perspective of future collaborations in this area, including the training in US diagnostic.

## Round table

The second and last day of the meeting concluded with a round table to answer the questions raised and to discuss the alternatives for control of the disease and how they can be implemented in Chile. The round table included the invited speakers Professor Marshall Lightowlers and Dr. Francesca Tamarozzi, plus the organizers of the event Dr. Sandra Cortes, Dr. Fernando Fredes, Dr. Marisa Torres, Dr. Gerardo Acosta-Jamett, Dr. Cristian Alvarez and also the president of the Chilean Society for Parasitology (SOCHIPA) Dr. Werner Apt.

## Final remarks

- Among the participants at the meeting it was evident during the round table discussions that a positive energy and willingness exist to implement actions against cystic echinococcosis. However, it is also clear that the different control activities in action in Chile were all using different approaches and that there was very limited communication between the groups involved.
- It was also evident that the control programmes face difficulties in gaining support for sufficient periods of time to ensure an enduring reduction in the incidence of human disease. It is clear to all the participants of the meeting that these control programmes have to be in action for a long period with constant reassessment of the results compared with the baseline of the situation of the disease established previous to the implementation of control measures.
- There is a need for the establishment of a national programme calling experts in the field to collaborate in the initiative. For this it is necessary to establish clear roles for the sectors involved: Universities, Government and privates, this should consider epidemiological, social and cultural aspects of what involves the establishment of this kind of initiative.
- Regarding the implementation of the vaccine: (i) due to the limitations in both the implementation and assessment of field trials that have been undertaken to date, we do not have sufficient, reliable data to be able to recommend wide-scale use of the EG95 vaccine for the control of CE; (ii) there is an urgent need for new, scientifically rigorous field assessments of the vaccine where vaccinations can be delivered reliably to most sheep and where sufficient numbers of sheep will be available for necropsy so that an accurate evaluation can be made of the vaccine’s usefulness as a CE control tool.
- It was agreed that abdominal ultrasound screening of human populations in Chile at risk of CE should be undertaken, allowing early treatment of cases and a more accurate determination of the prevalence of infection in the human population. An increased involvement by physicians in the control programmes in Chile is necessary as well as increased interaction with the veterinary sector involved in these programmes.
- In order to continue and enhance the systematic study of *E. granulosus* and its control in Chile, the Chilean organizers of the meeting proposed establishment of a “Cystic Echinococcosis Group in Chile” as an open and cooperative group which can coordinate activities, organise future meetings, prepare applications for research funding and continue the extension of activities and publishing the results obtained in research activities. The group would seek to collaborate with the government authorities in the design and improvements of the control plans for the disease in Chile.

## Abbreviations

INDAP: National Institute for the Development of Agriculture and Livestock (Instituto Nacional de Desarrollo Agropecuario)
ISP: National Institute for Public Health (Instituto de Salud Pública)
SAG: Animal Health Department (Servicio Agrícola y Ganadero)
CE: Cystic echinococcosis
RM: Metropolitan Region (Region Metropolitana)
US: ultrasound
MRI: Magnetic resonance imaging
CT: Computerised tomography
