# Erratum to: First meeting “Cystic echinococcosis in Chile, update in alternatives for control and diagnostics in animals and humans”

**DOI:** 10.1186/s13071-016-1874-x

**Published:** 2016-11-15

**Authors:** Cristian A. Alvarez Rojas, Fernando Fredes, Marisa Torres, Gerardo Acosta-Jamett, Juan Francisco Alvarez, Carlos Pavletic, Rodolfo Paredes, Sandra Cortés

**Affiliations:** 1 Centre for Animal Biotechnology, The University of Melbourne, Parkville, 3052 Victoria Australia; 2 Departamento de Medicina Preventiva Animal Facultad de Ciencias Veterinarias y Pecuarias, Universidad de Chile, Santiago, Chile; 3 Facultad de Medicina, Pontificia Universidad Católica de Chile, Santiago, Chile; 4 Instituto de Medicina Preventiva Veterinaria y Programa de Investigación Aplicada en Fauna Silvestre, Facultad de Ciencias Veterinarias, Universidad Austral de Chile, Casilla 567, Valdivia, Chile; 5 Servicio Agrícola Ganadero, Santiago, Chile; 6 Ministerio de Salud, Gobierno de Chile, Santiago, Chile; 7 Escuela de Medicina Veterinaria, Facultad de Ecología y Recursos Naturales, Universidad Andres Bello, Santiago, Chile; 8 Advanced Center for Chronic Diseases (ACCDiS), Facultad Medicina, Pontificia Universidad Católica de Chile, Santiago, Chile

## Erratum

Unfortunately, the original version of this article [1] contained an error. Within the author list, “Ministry of Health, SAG and other organizations attending the meeting” and “a list of collaborators” was erroneously included. The correct author list can be found here and has also been updated in the original article. The publisher would like to apologize for any inconvenience this oversight may have caused.
